# Antibiofilm activity of chlorhexidine and levofloxacin on pathogen causing orthopaedics implant-related infections

**DOI:** 10.1017/ash.2025.135

**Published:** 2025-09-03

**Authors:** Almarissa Ajeng Prameshwara, Vidyadhari Puspa Prawarni, Sayyidah Auliany Amminy, Kumala, Sholahuddin Rhatomy, Titik Nuryastuti

**Affiliations:** 1 Departement of Microbiology, Faculty of Medicine, Public Health and Nursing Gadjah MadaUniversity, Yogyakarta, Indonesia; 2 Indonesian Biofilm Research Collaboration Centre, Yogyakarta, Indonesia; 3 Department of Orthopaedics and Traumatology, Dr. Soeradji Tirtonegoro General Hospital, Klaten, Indonesia; Faculty of Medicine, Public Health, and NursingGadjah Mada University, Yogyakarta, Indonesia

**Keywords:** orthopaedics implant-related infection, biofilm, eradication, chlorhexidine, levofloxacin

## Abstract

**Objectives:** The incidence of hospital-acquired infections (HAIs) related to the formation of biofilms reaches 80% of the total cases of infection in the world. It can infect patients using invasive medical equipment such as orthopaedics implants. Biofilm-related implant orthopaedics infections account for approximately 65% of all bacterial infections. Once bacteria adhere to the implant, a bacterial community established biofilm that can enhance resistance to antibiotics up to 1000 times. Therefore, an appropriate strategy is needed to eradicate biofilm. Chlorhexidine as an antiseptic and levofloxacin as an antibiotic are often used in the orthopaedics’ setting. This study investigated in vitro antibiofilm activity of chlorhexidine and levofloxacin against bacterial isolates obtained from patient with implant orthopaedics-related infections. **Methods:** Ten clinical isolates of bacteria with strong biofilm-producer were collected from patients with orthopaedics implant-related infections including *Staphylococcus aureus* (n=2), *Staphylococcus haemolyticus* (n=1), *Serratia marcescens* (n=2), *Pseudomonas aeruginosa* (n=2), *Proteus mirabilis* (n=1), *Acinetobacter baumannii (*n=1*)* and *Klebsiella pneumoniae* (n=1). The inhibition and eradication activity of chlorhexidine and levofloxacin on biofilm growth were performed using microtiter broth dilution method in 96-well plates. **The minimum biofilm inhibitory concentration (MBIC) and minimum biofilm eradication concentration (MBEC) were determined using the MTT (3-(4-5- dimethylthiazol-2-yl)2,5-dyphenyl tetrazolium bromide) reduction assay.** **Results:** This study found that chlorhexidine inhibited the growth of Gram-positive bacterial biofilms by 80% with MBIC80 values ranged from 4-16 μg/ml and eradicated 80% of biofilm with MBEC80 value was 32 μg/ml. For Gram-negative bacterial biofilms, the ability of chlorhexidine to inhibit 80% of biofilm growth was indicated by MBIC80 values ranged from 8-16 μg/ml and to eradicate 80% of biofilm with MBEC80 values ranged from16-64 μg/ml. Meanwhile, levofloxacin can inhibit the growth of Gram- positive bacterial biofilms by 80% with MBIC80 values ranged from 1-4 μg/ml and can eradicate 80% of biofilm with MBEC80 values ranged from 16-32 μg/ml. For Gram-negative bacterial biofilms, the MBIC80 values of levofloxacin ranged from 1-16 μg/ml and the MBEC80 values ranged from 4-32 μg/ml. **Conclusions:** This result indicated that chlorhexidine and levofloxacin are potential to inhibit and eradicate bacterial biofilm. However, further studies need to be done for clinical evaluation.

